# Platinum nanoparticles on defect-rich nitrogen-doped hollow carbon as an efficient electrocatalyst for hydrogen evolution reactions

**DOI:** 10.1039/c9ra09662k

**Published:** 2020-01-03

**Authors:** Yapeng Cheng, Meiling Fan, Weiran Lin, Zhiwei Zhang, Haining Zhang

**Affiliations:** State Key Laboratory of Advanced Technology for Materials Synthesis and Processing, Wuhan University of Technology Nr. 122 Luoshi Rd Wuhan 430070 China linwr@tsinghua.edu.cn haining.zhang@whut.edu.cn; The Fundamental Industry Training Center, Tsinghua University Beijing 100084 China

## Abstract

Design and synthesis of efficient electrocatalysts with low usage of precious metal and of high stability are essential for their practical applications in hydrogen evolution reactions. In this work, we synthesize an electrocatalyst through the deposition of platinum nanoparticles on defect-rich nitrogen-doped hollow carbon derived from surface-attached poly(4-vinylpyridine) monolayers. The platinum nanoparticles with an average diameter of about 1.8 nm are well dispersed on the outer surface of the pre-synthesized carbon material and the platinum loading is about 8.6 wt%. The mass activity of the as-synthesized catalyst under an overpotential of 55 mV is about 5.0 A mg_Pt_^−1^, about 4.93 times higher than that of commercial Pt/C catalysts. Moreover, the synthesized catalyst is also more electrochemically stable than commercial Pt/C catalysts as evidenced by continuous cyclic voltammetry and chronoamperometric response measurements.

## Abstract 2

Design and synthesis of efficient electrocatalysts with low usage of precious metal and of high stability are essential for hydrogen evolution reaction in their practical applications.

## Graphical abstract

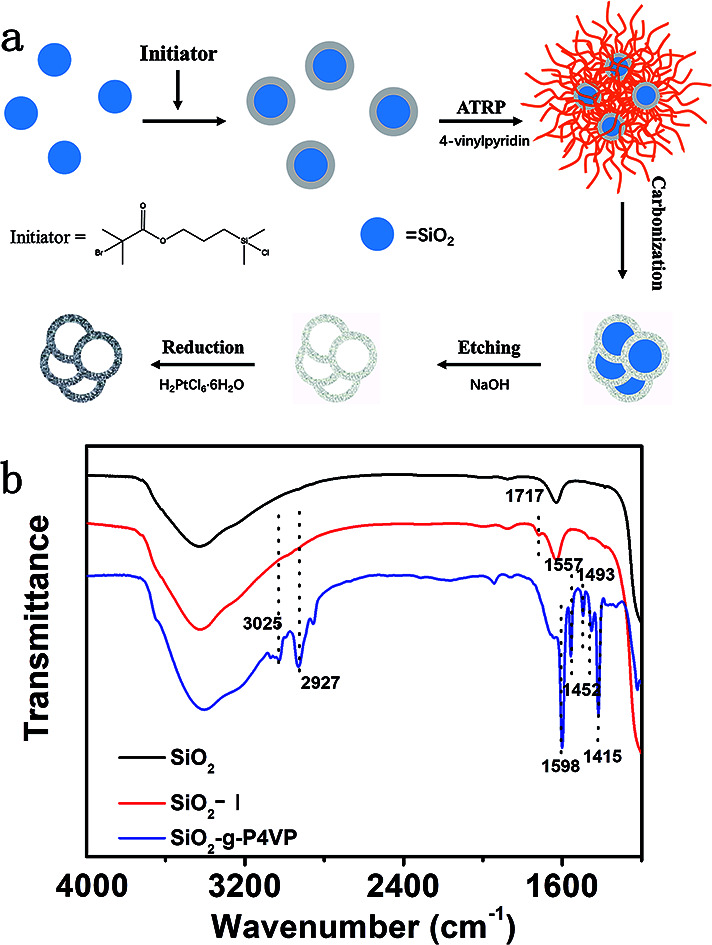

## Introduction

Sustainable and clean energy for growing market demands is always a worldwide hot topic to address the environmental problems caused by consumption of fossil fuels.^1–3^ As a promising clean energy, hydrogen energy has attracted extensive attention due to its high mass-specific energy density and zero emissions.^4–7^ Currently, hydrogen generation mostly relies on the reformation of natural gas or methane, which still results in carbon emission during the hydrogen generation process. In contrast, hydrogen generation through electrocatalytic water splitting has been realized as an efficient and clean technology, but the application of noble-metal electrocatalysts, mainly Pt-based materials, for the hydrogen evolution reaction (HER) leads to high costs, hindering widespread applications.^5,8,9^ Despite the fact that numerous research works have been conducted for the development of non-noble metal HER electrocatalysts, Pt-based nanomaterials are still the most successful and commonly used catalysts for the HER.^10^ Apart from the cost, the aggregation of Pt nanoparticles during operation can result in a drastic reduction in catalytic performance, which in turn affects the durability of the catalysts and the accordingly assembled devices. Thus, development of electrocatalysts with relatively low Pt loading, high utilization efficiency, and improved durability is of great importance for the HER.^11–15^

Carbon-based materials have been widely studied as supporting materials to enhance the electrochemical properties of Pt nanoparticles since they exhibit excellent electrochemical properties and stability, and are of low cost.^16,17^ Recently, nanostructured carbon materials with designed defects, particularly caused by heteroatom doping, have been realized as promising electrocatalysts or supporting materials for the improvement of catalytic activity.^18,19^ Introduced heteroatoms can donate electrons to neighboring carbon atoms and can change the nucleation and growth kinetics of metallic nanoparticles to reduce the particle size, to improve the distribution of the formed nanoparticles, and to protect the metallic nanoparticles against agglomeration.^8,20–24^ As an example, Timothy *et al.* reported that N-implanted defects have a strong force and intrinsic effect for nucleation and tethering Pt clusters respectively.^20^

Of the various nanostructured carbon-based materials, hollow porous carbon has been recognized as a promising support matrix for Pt nanoparticles to enhance their electrocatalytic activity due to the properties of low density, large surface area, fast mass transport, and enough active centers to anchor Pt nanoparticles.^25^ The often-applied approach to synthesize hollow porous carbon is the so-called “hard template” method, in which polymeric precursors and hard templates (typically silica nanoparticles) are physically mixed and pyrolyzed at high temperature, followed by removal of the templates. However, the relatively weak interaction between precursor and template and the possible aggregation of templates during the pyrolysis process make the pore size and pore size distribution of the final carbon products less controllable. Accordingly, the further deposition of metal nanoparticles and the electrocatalytic activity of the final catalysts remain unsatisfactory. Therefore, a sophisticated synthetic strategy is required to develop a superior hollow porous structure rich in active sites as a carbon support for the uniform dispersion and anchoring of Pt nanoparticles.

Herein, we present a unique strategy to synthesize a defect-rich hollow porous carbon with an abundant source of heteroatom dopants as a support material for the deposition of Pt nanoparticles. For this, surface-attached polymer monolayers formed through atom transfer radical polymerization (ATRP) were applied as precursors.^26^ After the formation of hollow porous carbon by pyrolysis and removal of silica templates, the Pt nanoparticles were finally deposited on the surface of the carbon material to form the final electrocatalyst for the HER. It is expected that the hollow porous structure and the possible anchoring sites for Pt nanoparticles can possibly improve the HER electrocatalytic activity and stability of Pt-based catalysts compared to commercial Pt/C catalysts.

## Experimental section

### Materials

4-Vinylpyridine, acryl alcohol, and bromoisobutyryl bromide were purchased from Sigma-Aldrich. *N*,*N*-Dimethylformamide (DMF), triethylamine, and cuprous chloride (CuCl) were received from Sinopharm Chemical Reagent. Dichloromethane, tris[2-(dimethylamino)ethyl]amine (Me_6_TREN), and dimethylchlorosilane were purchased from Alfa Aesar. Silica nanoparticles (30 nm, 99.5%) were purchased and used without further purification. Deionized water (18.2 MΩ cm) was obtained through an ultrapure water generator (Ulup, China). Toluene was distilled over sodium using benzophenone as an indicator and stored with molecular sieves. The chlorosilane-functionalized initiator molecules and the initiator-modified silica nanoparticles for ATRP were synthesized according to the literature.^27^

### Synthesis of surface-attached poly(4-vinylpyridine) on silica nanoparticles

0.5 g initiator-modified silica nanoparticles and 10 mL 4-vinylpyridine were dispersed in 6.0 mL of DMF under protection of nitrogen atmosphere. After degassing under vacuum through three freeze–thaw cycles, a solution of CuCl (0.016 g) and Me_6_TREN (1.0 mL) in 10 mL DMF was added to the dispersion and the mixture was heated to 90 °C for polymerization. The polymerization was carried out for 12 h. After collection by centrifugation, the products were extensively rinsed with ethanol, followed by Soxhlet extraction and drying under vacuum. The obtained poly(4-vinylpyridine)-grafted silica nanoparticles were denoted as SiO_2_-g-P4VP.

### Synthesis of defect-rich nitrogen-doped hollow carbon (DNHC)

Typically, 1.0 g of SiO_2_-g-P4VP was placed in a quartz tube furnace under an atmosphere of nitrogen. After continually purging with N_2_ for half an hour, the sample was heated to 900 °C with a ramp rate of 5.0 °C min^−1^ and kept at 900 °C for 2 h. After cooling to room temperature, the powder was collected and ground for 20 min. The sample was then immersed into 5.0 mol L^−1^ NaOH aqueous solution for 48 h to remove the silica templates. After being extensively washed with deionized water until a neutral eluent was reached and drying in a vacuum oven at 60 °C, the final product was obtained and was denoted as DNHC.

### Synthesis of Pt@DNHC

The Pt@DNHC composite catalyst was fabricated by reduction of H_2_PtCl_6_ using ethylene glycol as reductant in the presence of DNHC. Briefly, to a suspension of DNHC (60 mg) in 60 mL ethylene glycol, 10 mL of H_2_PtCl_6_ (7.74 mmol L^−1^ in ethylene glycol) was added dropwise under vigorous stirring using a magnetic stirrer. After 30 min stirring, the pH value of the mixture was adjusted to about 10.0 using 1.0 mol L^−1^ NaOH solution in ethylene glycol and the mixture was placed in an oil bath pre-heated to 135 °C. After 4 h reaction, the mixture was allowed to cool down to room temperature. The final product, Pt@DNHC, was obtained by centrifugation and extensively washing with ethanol, followed by drying using a lyophilizer. The theoretical Pt loading was calculated according to the amount of added H_2_PtCl_6_ solution but it should be noted that not all of the Pt could be reduced on the surface of DNHC.

### General characterization

The chemical composition of samples was determined from Fourier transform infrared (FTIR) spectra obtained using a 60SXB spectrometer (Nicolet) with a resolution of 4 cm^−1^. X-ray diffraction (XRD) patterns were recorded using an X-ray diffractometer (D/MAX-RB RU-200B, Japan) with filtered Cu Kα radiation (*λ* = 1.5406 Å, operating at 45 kV and 200 mA) to evaluate the crystallographic structure. Raman spectra were collected using a Jobin Yvon LabRam HR800 instrument with a He–Ne laser of 532.15 nm. Scanning electron microscopy (SEM) and transmission electron microscopy (TEM) were applied to analyze the more detailed structure of samples. High-resolution TEM, energy dispersive X-ray spectroscopy elemental mapping, and high-angle annular dark-field scanning TEM (HAADF-STEM) were conducted using a JEM-2100F with an acceleration voltage of 200 kV to qualitatively analyze the local composition of the catalysts. X-ray photoelectron spectroscopy (XPS) was carried out with a VG Multilab2000X to determine the surface elemental composition and bonding configuration of the prepared samples. Surface area of samples was calculated from N_2_ adsorption/desorption isotherms performed with a Micromeritics ASAP 2020 instrument at 77 K using Brunauer–Emmett–Teller (BET) and Barrett–Joyner–Halenda (BJH) models to calculate the specific surface area and pore size, respectively. The bulk content of Pt in samples was estimated by inductively coupled plasma-atomic emission spectroscopy (Vista MPX).

### Electrochemical measurements

All electrochemical tests were carried out with a CHI630E workstation at room temperature with a typical three-electrode system, in which a graphite rod (Alfa Aesar, 99.9995%) and a Pt foil (for electrochemical active surface area, ECSA) were used as the counter electrode, catalyst-coated glassy carbon electrode (GCE; diameter = 3 mm) as working electrode, and KCl-saturated calomel electrode as reference electrode which was corrected with reversible hydrogen electrode (RHE) in all measurements. A 0.5 mol L^−1^ N_2_-saturated H_2_SO_4_ aqueous solution was used as electrolyte for HER and all polarization curves are used for *iR* correction by electrochemical impedance spectroscopy from 10 kHz to 0.1 Hz at −40 mV (*vs.* RHE). The *iR*-corrected potential was obtained by the equation:*E*_*iR*-corrected_ = *E* − *iR*where *E*_*iR*-corrected_, *E*, *i* and *R* are the *iR*-corrected potential, the original potential, the corresponding current and the internal resistance respectively.

### Catalyst ink preparation

5 mg of catalyst powder and 40 μL of Nafion dispersion (5 wt% in isopropanol and water mixture with a volume ratio of 9 : 1) were added into a mixed solution of 100 μL deionized water and 860 μL isopropanol. The mixture was ultrasonicated for 30 min to form a homogeneous suspension. 4 μL catalyst dispersion from the prepared homogeneous inks was then carefully dropped onto a GCE (diameter = 3 mm) with a catalyst loading of 0.286 mg cm^−2^ and dried in air. Commercial Pt/C was tested in the same way.

### HER test

Cyclic voltammetry (CV) was operated with a scan rate of 0.1 V s^−1^ for at least 20 cycles to activate the catalyst and then the electrocatalytic activity was measured by linear sweep voltammetry in 0.5 M H_2_SO_4_ solution. The Tafel slopes as a significant factor to judge the electrochemical behavior were derived from the polarization curves and calculated based on the following Tafel equation:*η* = *b* log(*j*) + *a*where *η*, *a*, *b* and *j* are the overpotential, the Tafel constant, the Tafel slope and the current density.

### Stability test

Accelerated durability tests were performed in 0.5 M H_2_SO_4_ solution by applying cyclic sweeps between 0 and 60 mV (*vs.* RHE) at a sweep rate of 100 mV s^−1^ for 5000 cycles. For further TEM characterizations, the catalysts after the durability tests were collected from the GCE by sonicating the electrode in ethanol. In addition, the *I*–*t* chronoamperometric response was also recorded at a current density of 10 mA cm^−2^ for 10 h in 0.5 M H_2_SO_4_ solution to evaluate the long-term durability of catalysts.

### ESCA

ECSA is an important index to judge the electrochemical activity of catalysts. CV was also applied with a scan rate of 0.1 V s^−1^ for at least 20 cycles at a voltage of 0–1.2 V (*vs.* RHE) to activate the catalyst and operated with a scan rate of 0.01 V s^−1^ for 2 cycles in N_2_-saturated 0.5 M H_2_SO_4_ to obtain CV curves. ECSA was calculated through the formulas:*Q*_H_ = *S*/*V*
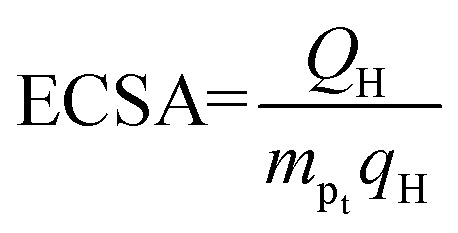
where *S*, *V*, *m*_Pt_ and *q*_H_ represent integral area in hydrogen adsorption zone, scan rate, Pt loading in GCE and a constant (0.21 mC cm^−2^) respectively.

## Results and discussion

**Fig. 1:**
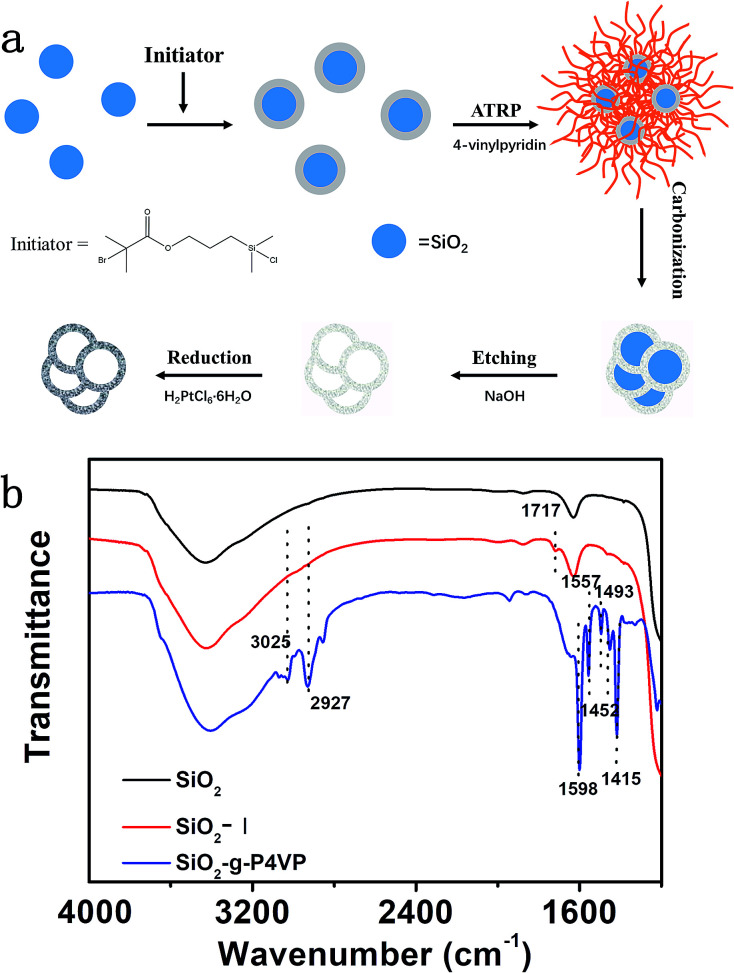
(a) Schematic illustration of the synthesis of Pt@DNHC. (b) FTIR spectra of bare silica, initiator-modified silica, and polymer-grafted silica nanoparticles as indicated in the figure. Dashed lines in (b) are guides to the eye.

**Fig. 2:**
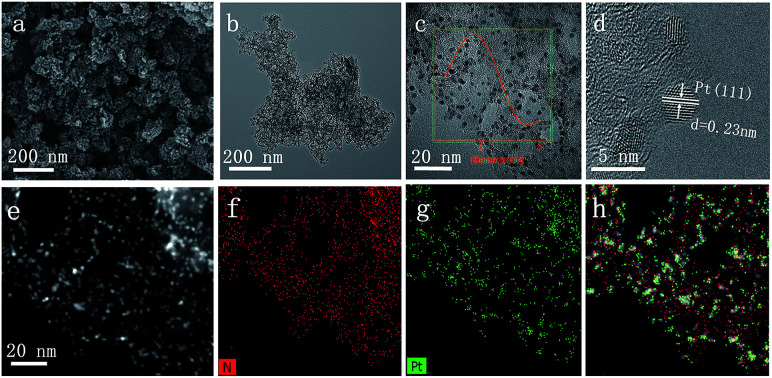
SEM image (a), TEM image (b), high-resolution TEM images (c and d), and HAADF-STEM image (e) of Pt@DHPC, and the corresponding elemental mapping of N atoms (f), Pt atoms (g) and the combined results (h). The inset in (c) is the size distribution of Pt nanoparticles estimated from the image.

The designed Pt@DNHC catalyst was synthesized by deposition of Pt nanoparticles onto pre-formed DNHC derived from surface-grafted poly(4-vinylpyridine) monolayers (SiO_2_-g-P4VP), as schematically shown in Fig. 1a. To synthesize DNHC, silica nanoparticles were used as templates for the generation of a hollow structure and surface-attached P4VP monolayers act as both nitrogen and carbon sources.^27^ FTIR spectra (Fig. 1b) were recorded to confirm the self-assembly of initiator and the attachment of polymer chains on the silica nanoparticles. Compared to the FTIR spectrum of bare silica nanoparticles, the absorption band appearing at about 1717 cm^−1^ is assigned to the stretching vibration of C=O bond, confirming the successful immobilization of initiator molecules on the surface of SiO_2_. After surface-initiated ATRP, the observed new absorption bands in the FTIR spectrum of the resulting sample at 1598, 1557, 1452, and 1415 cm^−1^ correspond to C=C and C=N bonds of pyridine ring, suggesting the P4VP chains are attached to the surface of silica nanoparticles. The designed DNHC was then synthesized by carbonization of SiO_2_-g-P4VP, followed by removal of silica templates. The Pt nanoparticles were finally deposited on the surface of DNHC by reduction of H_2_PtCl_6_ at 135 °C using ethylene glycol as reductant. The Pt loading in the synthesized Pt@DNHC is 8.6 wt%, as quantitatively determined by inductively coupled plasma-atomic emission spectroscopy. The morphology of Pt@DNHC and the size distribution of Pt nanoparticles were first investigated using electron microscopy, as shown in Fig. 2. SEM image (Fig. 2a) revealed that the formed Pt@DNHC has a hollow appearance and TEM image (Fig. 2b) suggested a 3D hollow structure of Pt@DNHC with abundant broken edges which may provide active sites to promote the electrochemical performance.^28^ In addition, the average inner diameter of the synthesized DNHC is about 25 nm, slightly smaller than that of the silica templates, indicating the slight shrinkage of the formed carbon material during the process of removal of the silica templates. From the high-resolution TEM image of Pt@DNHC (Fig. 2c), it can be clearly seen that the formed Pt nanoparticles are well distributed on the disordered carbon layer of DNHC and the average diameter of Pt nanoparticles is centered at 1.83 nm (inset in Fig. 2c), suggesting the Pt nanoparticles are surrounded with defect-rich structure on the doped carbon substrate. Moreover, the observed interlayer spacing of 0.23 nm corresponds to the (111) planes of Pt nanoparticles (Fig. 2d), further confirming the successful reduction to form Pt nanoparticles. Fig. 2e presents the HAADF-STEM image of Pt@DNHC and the corresponding elemental mapping results are displayed in Fig. 2f–h. The formed Pt nanoparticles are well distributed around the uniform doped nitrogen atoms in the defect-rich hollow porous carbon which may contribute to high energy vacancies, provided by N-modified carbon atoms, for the growth of Pt nanoparticles.

**Fig. 3:**
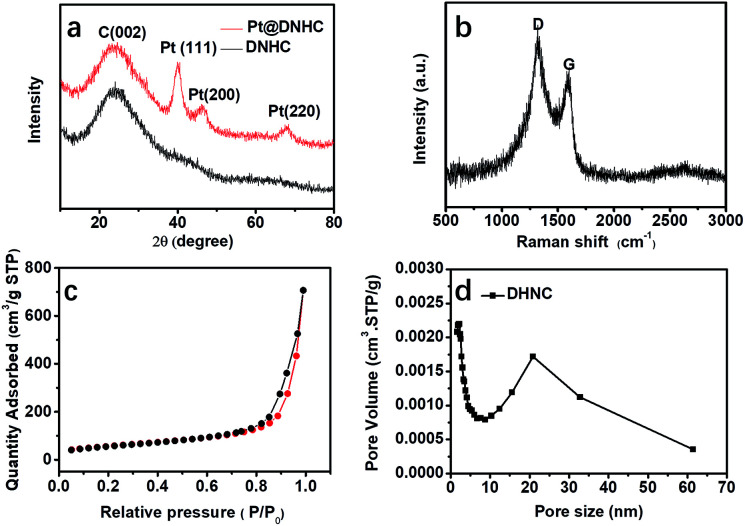
XRD pattern (a), Raman spectrum (b), nitrogen adsorption–desorption isotherm (c), and pore size distribution (d) of Pt@DNHC.

To get further insight into the crystalline structures, XRD measurement was performed to analyze the carbon matrix and the deposited Pt nanoparticles, as shown in Fig. 3a. Compared to the XRD pattern of DNHC, new diffraction peaks centered at 39.91°, 46.21° and 67.78° were observed in the XRD pattern of Pt@DNHC, corresponding to the (111), (200) and (220) planes of cubic Pt, respectively. In addition, the average size of the formed Pt nanoparticles was also estimated to be about 2.46 nm by the Scherrer equation using the diffraction peak of (111), which is larger than that derived from TEM images due to the difference in the calculated area. Fig. 3b displays the Raman spectrum of Pt@DNHC. It is apparent that the intensity of the G band at 1350 cm^−1^ is much higher than that of the D band at 1590 cm^−1^. The intensity ratio of 1.28 for the synthesized DNHC material indicates a highly defective structure, which provides nucleation energy offered by the difference between a defect site and defect-free area.^20^ Nitrogen adsorption–desorption isotherm of Pt@DNHC was recorded as shown in Fig. 3c and the accordingly derived pore size distribution is plotted in Fig. 3d. The observed type IV isotherm suggested the existence of mesopores and the average pore diameter is about 25 nm, which is in good agreement with TEM observations. In addition, the calculated BET surface area of Pt@DNHC is about 203 m^2^ g^−1^. Such high surface area provides abundant sites to anchor Pt nanoparticles and the porous structure benefits chemical product transmission.^23,29,30^

**Fig. 4:**
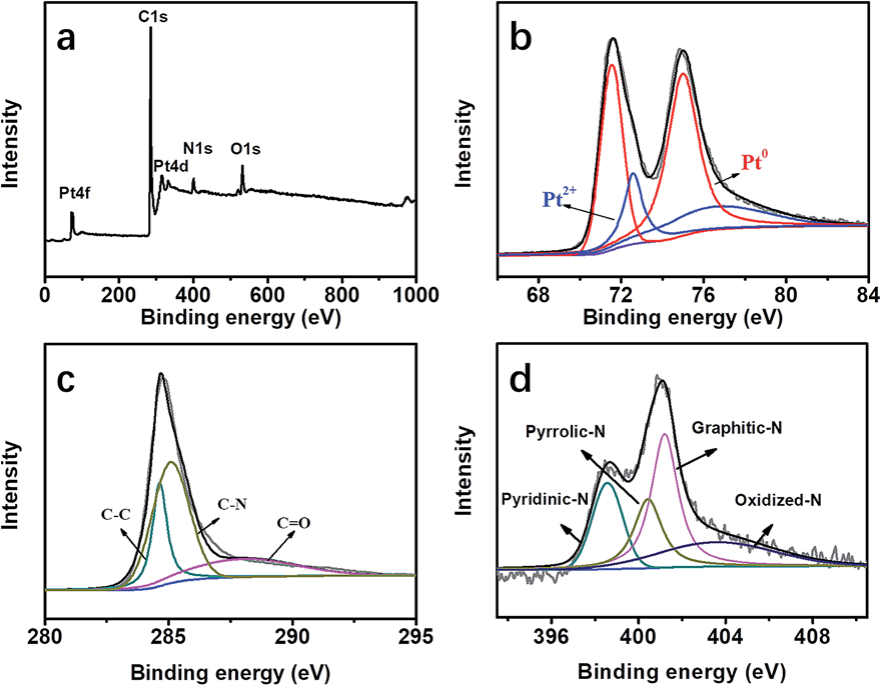
(a) Full XPS survey of Pt@DNHC. High-resolution XPS spectra and the deconvoluted results of Pt 4f (b), C 1s (c) and N 1s (d) in the Pt@DNHC catalyst.

XPS measurements were carried out to understand the chemical environment and bonding states of the atoms in the formed Pt@DNHC, as shown in Fig. 4. From the full XPS survey of Pt@DNHC (Fig. 4a), the peaks for Pt at 72.5 and 316.0 eV, C at 285.9 eV, N at 401.4 eV, and O at 533.1 eV were clearly observed. Fig. 4b shows the high-resolution XPS spectrum of Pt 4f, which displayed the characteristic peaks of Pt 4f_7/2_ at 71.5 eV and Pt 4f_5/2_ at 75.1 eV. The high-resolution Pt 4f spectrum was deconvoluted into two pairs of doublets. The doublet at 71.6 eV and 75.0 eV represented the bulk of Pt and the one at 72.6 eV and 76.7 eV corresponds to the Pt^2+^ state due to the complexation with N atoms.^13^ From the deconvolution results of the high-resolution XPS spectrum of C 1s (Fig. 4c), the peak at 285.2 eV corresponds to C–N, indicating that the nitrogen atoms are doped into the carbon frameworks. The high-resolution XPS spectrum of N 1s (Fig. 4d) could be deconvoluted into four chemical components at 398.8, 400.5, 401.1, and 403.8 eV, corresponding to pyridinic-N, pyrrolic-N, graphitic-N, and oxidized-N, respectively.^15^ In addition, the content of pyridinic-N is about 37 at% of the overall doped nitrogen atoms, which can modify the electronic structure of neighboring carbon atoms anchoring Pt nanoparticles and limiting their aggregation.

Hydrogen production through electrochemical water splitting is of great importance for hydrogen energy-based applications. Since Pt is still the optimal choice for HER catalysis,^31^ the electrochemical HER performance of the synthesized Pt@DNHC catalyst was finally evaluated in 0.5 mol L^−1^ H_2_SO_4_ with a scan rate of 5 mV s^−1^. For comparison, the electrochemical properties of commercial Pt/C catalysts (20 wt% of Pt) were also investigated.

**Fig. 5:**
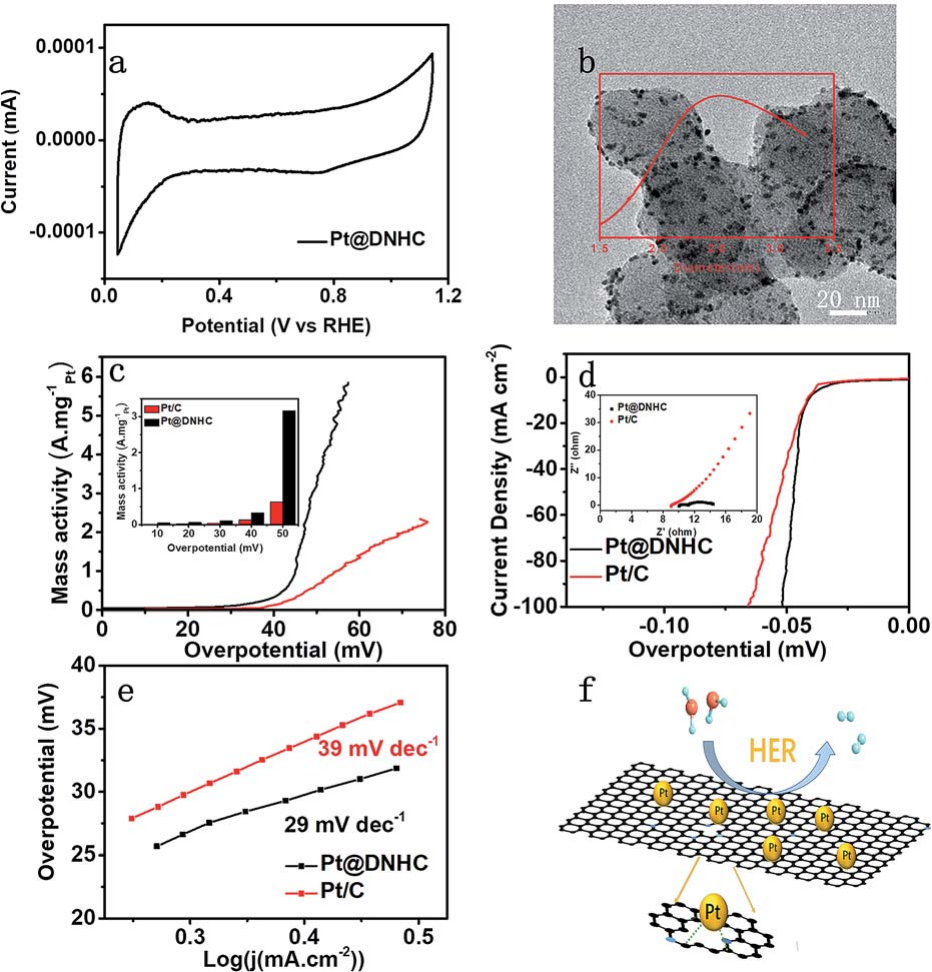
(a) CV curve of Pt@DNHC in 0.5 mol L^−1^ H_2_SO_4_ aqueous solution with a scan rate of 5 mV s^−1^. (b) TEM image of commercial Pt/C (20 wt% of Pt) and the particle size distribution derived from the image (inset). (c) Mass activity of Pt@DNHC and commercial Pt/C catalysts under different overpotentials. (d) HER polarization curves of Pt@DNHC and Pt/C. (e) Tafel plots of Pt@DNHC and commercial Pt/C catalysts. (f) Schematic process of HER using Pt@DNHC catalyst. The insets in (c) and (d) show the mass activity of Pt@DNHC and Pt/C at different overpotential and electrochemical impedance spectroscopy Nyquist plots of Pt@DHNC and Pt/C at −0.040 V (*vs.* RHE) in H_2_SO_4_ respectively.

For electrocatalysts, ECSA is one of the important parameters related to catalytic activity. The ECSA of the synthesized Pt@DNHC catalyst was determined from the CV curve in 0.5 mol L^−1^ H_2_SO_4_ (Fig. 5a) to be 63.7 m^2^ g^−1^ whereas it is about 44.4 m^2^ g^−1^ for the commercial Pt/C catalysts with 20 wt% of Pt. It should be also noted that the content of Pt in Pt@DNHC is about 8.6 wt%. High ECSA of Pt@DNHC contributes to exposing more active sites and highly active crystal faces to adsorb H^+^ ions with excellent efficiency. To understand this difference, TEM image of a commercial Pt/C catalyst was obtained and is shown in Fig. 5b. The average diameter of Pt nanoparticles in Pt/C catalysts is about 2.84 nm, much larger than that in Pt@DNHC (1.83 nm from Fig. 2c). This result suggests that the difference in ECSA values between Pt@DNHC and commercial Pt/C catalysts resulted from the different particle size of Pt nanoparticles.

To investigate the HER performance of the synthesized Pt@DNHC catalyst, the polarization curves of Pt@DNHC, commercial Pt/C, and pristine DNHC are plotted in Fig. 5d. It is apparent that the pristine DNHC has no activity for HER whereas both Pt@DNHC and commercial Pt/C catalysts exhibit good electrocatalytic activity towards HER as evidenced by the onset potential. Moreover, Pt@DNHC exhibits better HER performance than the commercial Pt/C, as evidenced by the lower overpotential of Pt@DNHC than that of commercial Pt/C at the same current density. For example, an overpotential of 41 mV is required to generate a current density of 10 mA cm^−2^ for Pt@DNHC and commercial Pt/C under the same condition, which was compared with some state-of-the-art works in Table 1. Considering the Pt loading in the catalyst, the mass activity, which refers to the generated current normalized to Pt loading under certain overpotential of both Pt@DNHC and commercial Pt/C catalysts, is displayed in Fig. 5c. It is evident that the mass activity of Pt@DNHC is much higher than that of commercial Pt/C and the difference is more pronounced at higher overpotentials. Specifically, the mass activity of Pt@DNHC at an overpotential of 40 mV is about 2.54 times higher than that of Pt/C catalyst (0.131 A mg_Pt_^−1^) whereas under an overpotential of 55 mV, the mass activity of Pt@DNHC reaches 3.38 A mg_Pt_^−1^, 5.32 times higher than that of commercial Pt/C catalyst. The great mass activity of Pt@DNHC for HER could be possibly attributed to the small Pt nanoparticles inducing large ECSA and the possible synergetic effect of the nitrogen atoms.^20^ Tafel plots were further applied to get further insight into the mechanism of HER, as shown in Fig. 5e. It is evident that the resulting Tafel slopes are 29 and 39 mV dev^−1^ for Pt@DNHC and commercial Pt/C catalysts respectively, suggesting that the HER process for both catalysts under acidic conditions follows a Volmer−Heyrovsky mechanism.^32,33^ Moreover, the smaller Tafel slope for Pt@DNHC compared to Pt/C indicates that the electrode reaction kinetics using Pt@DNHC catalyst is also faster than that using commercial Pt/C catalyst. Fig. 5f displays the brief HER process using Pt@DNHC as catalyst. Apart from the great electrocatalytic activity towards HER induced by the well-distributed small Pt nanoparticles, the designed Pt@DNHC catalyst is also expected to be highly stable due to the interaction between the N-modified carbon atoms and the Pt nanoparticles.^14^

**Table 1:** Comparison of Pt@DNHC in this work and state-of-the-art literature

Sample	Overpotential at 10 mA cm^−2^ (mV)	Electrolyte	Reference
Pt/WS2	80	0.5 M H_2_SO_4_	24
Pt_1_@Fe–N–C	60	0.5 M H_2_SO_4_	32
Pt/NPC	21.7	0.5 M H_2_SO_4_	13
Pt–Ni_3_N/Ni@C	47	0.5 M H_2_SO_4_	34
Pt/CNTs-ECR	34	0.5 M H_2_SO_4_	35
Pt@DNHC	41	0.5 M H_2_SO_4_	This work

**Fig. 6:**
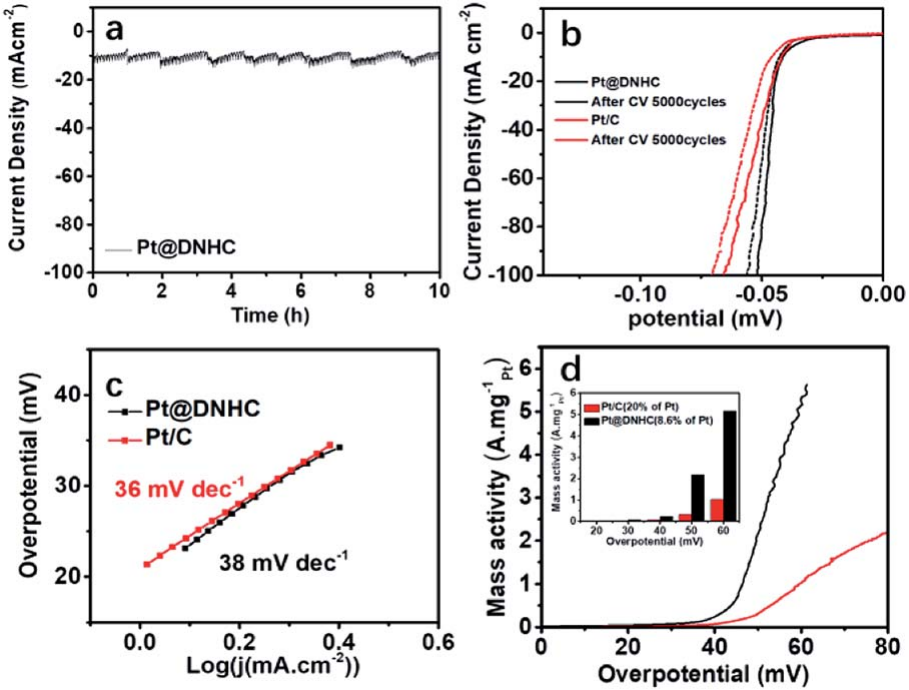
(a) *I*–*t* chronoamperometric response at a current density of 10 mA cm^−2^ in 0.5 mol L^−1^ aqueous H_2_SO_4_. (b) Polarization curves of Pt@DNHC and commercial Pt/C before and after 5000 continuous CV cycles. (c) Tafel plots and (d) mass activity of Pt@DNHC and commercial Pt/C catalysts after 5000 continuous CV cycles. The CV cycles were scanned in 0.5 mol L^−1^ aqueous H_2_SO_4_ from 0 to 0.05 V with a scan rate of 100 mV s^−1^. The inset in (d) is the mass activity of Pt@DNHC and Pt/C at different overpotentials.

We thus evaluated the stability of the synthesized Pt@DNHC catalyst through I-t chronoamperometric response at a current density of 10 mA cm^−2^ in 0.5 mol L^−1^ H_2_SO_4_ solution, as displayed in Fig. 6a. It can be clearly seen that the current density has no obvious change after the successive operation of HER for 10 h, indicating the superior stability of the synthesized Pt@DNHC. Such an electrochemical stability of Pt@DNHC could be possibly attributed to the immobilization of Pt nanoparticles to the nitrogen sites of DNHC, on which Pt nanoparticles nucleate and are anchored strongly.

A comparison of the stability of Pt@DNHC with that of commercial Pt/C catalyst was also made to evaluate its practical applications. Fig. 6b shows the HER polarization curves of Pt@DNHC and commercial Pt/C catalysts after 5000 continuous CV cycles in the range of 0 and 0.06 V at room temperature in 0.5 mol L^−1^ H_2_SO_4_ aqueous solutions with a scan rate of 100 mV s^−1^. It is apparent that the HER performance decay of Pt@DNHC is less significant compared to commercial Pt/C catalyst as evidenced by the shift of onset potential and overpotential at a certain current density for both samples. Specifically, the onset potential had no obvious shift and the overpotential at a current density of 20 mA cm^−2^ was negatively shifted by 1 mV for Pt@DNHC after 5000 continuous CV cycles, whereas both onset potential and overpotential at 10 mA cm^−2^ were negatively shifted by 5.0 mV for commercial Pt/C catalyst, indicating the higher stability of Pt@DNHC compared to commercial Pt/C catalyst. In addition, Tafel slopes for both Pt@DNHC and Pt/C catalysts (Fig. 6c) after 5000 continuous CV cycles are close to the initial values, suggesting no obviously significant changes in HER mechanism and process. Moreover, the mass activities of Pt@DNHC and commercial catalysts after 5000 continuous CV cycles are plotted in Fig. 6d for a direct comparison of the performance decay. It was observed that the mass activity of Pt@DNHC is about 5.57 times higher than that of commercial Pt/C and the difference in mass activity for Pt@DNHC and Pt/C became larger than the initial values, suggesting that the HER performance decay of commercial Pt/C catalyst is more marked than that of the synthesized Pt@DNHC.

**Fig. 7:**
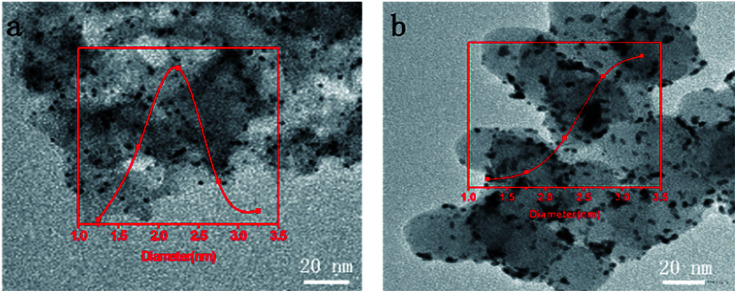
TEM images of Pt/DNHC (a) and commercial Pt/C (b) catalysts after 5000 continuous CV cycles in 0.5 mol L^−1^ H_2_SO_4_ aqueous solution. The insets are statistical results of the average diameters from the respective TEM images.

It was hypothesized that the superior electrochemical stability of Pt@DNHC could be attributed to the immobilization of Pt nanoparticles on the surface of DNHC through modification with the doping nitrogen atoms, which in turn prevents the deposited Pt nanoparticles from aggregation. To confirm this hypothesis, TEM images of Pt@DNHC and commercial Pt/C catalysts after 5000 continuous CV cycles are displayed in Fig. 7. Visual inspection has allowed drawing the qualitative conclusion that the size of Pt nanoparticles remains quite uniform for Pt@DNHC and the aggregation of Pt nanoparticles is clearly observed for commercial Pt/C catalyst after 5000 continuous CV cycles. The average diameter of Pt nanoparticles for Pt@DNHC increased from the initial value of 1.8 to 2.2 nm after 5000 continuous CV cycles whereas it increased from 2.8 to 3.9 nm for commercial Pt/C catalyst, resulting in the more stable HER performance of Pt@DNHC than that of commercial Pt/C catalysts.

## Conclusions

A platinum-based electrocatalyst for the HER was synthesized through *in situ* deposition of Pt nanoparticles on the surface of a pre-synthesized DNHC support. To have better porous structure of the support and good distribution of the deposited Pt nanoparticles, surface-attached poly(4-vinylpyridine) monolayers on silica nanoparticles *via* surface-attached ATRP technique were applied as precursor and the silica templates were finally removed after carbonization. Benefiting from the porous structure and the good distribution of nitrogen, the deposited small Pt nanoparticles were well distributed on the carbon-based support with relatively low Pt loading of about 8.6 wt% due to the possible strong interaction between Pt and high-energy vacancies provided by the N-implemented defects. Such a small particle size of the deposited Pt nanoparticles resulted in a large ECSA of the synthesized catalyst compared to a commercial Pt/C catalyst with Pt loading of 20 wt%. Accordingly, the synthesized catalyst exhibited a better electrocatalytic activity and electrochemical stability for the HER compared with the commercial Pt/C catalysts. The results described demonstrate that the composite of Pt nanoparticles on DNHC support could be an effective and practical electrocatalyst for hydrogen evolution reaction with reduced Pt loading and improved stability compared with commercial Pt/C catalysts.

## Conflicts of interest

The authors declare no competing financial interest.

## Supplementary Material
